# ChatGPT: is it good for our glaucoma patients?

**DOI:** 10.3389/fopht.2023.1260415

**Published:** 2023-11-16

**Authors:** Gloria Wu, David A. Lee, Weichen Zhao, Adrial Wong, Sahej Sidhu

**Affiliations:** ^1^ Department of Ophthalmology, University of California San Francisco, San Francisco, CA, United States; ^2^ Department of Ophthalmology, McGovern Medical School, University of Texas Health Science Center, Houston, TX, United States; ^3^ Department of Biological Sciences, University of California Davis, Davis, CA, United States; ^4^ Department of Biology, Santa Clara University, Santa Clara, CA, United States

**Keywords:** glaucoma, ophthalmology, ChatGPT, artificial intelligence, patient education

## Abstract

**Purpose:**

Our study investigates ChatGPT and its ability to communicate with glaucoma patients.

**Methods:**

We inputted eight glaucoma-related questions/topics found on the American Academy of Ophthalmology (AAO)’s website into ChatGPT. We used the Flesch–Kincaid test, Gunning Fog Index, SMOG Index, and Dale–Chall readability formula to evaluate the comprehensibility of its responses for patients. ChatGPT’s answers were compared with those found on the AAO’s website.

**Results:**

ChatGPT’s responses required reading comprehension of a higher grade level (average = grade 12.5 ± 1.6) than that of the text on the AAO’s website (average = 9.4 grade ± 3.5), (0.0384). For the eight responses, the key ophthalmic terms appeared 34 out of 86 times in the ChatGPT responses vs. 86 out of 86 times in the text on the AAO’s website. The term “eye doctor” appeared once in the ChatGPT text, but the formal term “ophthalmologist” did not appear. The term “ophthalmologist” appears 26 times on the AAO’s website. The word counts of the answers produced by ChatGPT and those on the AAO’s website were similar (*p* = 0.571), with phrases of a homogenous length.

**Conclusion:**

ChatGPT trains on the texts, phrases, and algorithms inputted by software engineers. As ophthalmologists, through our websites and journals, we should consider encoding the phrase “see an ophthalmologist”. Our medical assistants should sit with patients during their appointments to ensure that the text is accurate and that they fully comprehend its meaning. ChatGPT is effective for providing general information such as definitions or potential treatment options for glaucoma. However, ChatGPT has a tendency toward repetitive answers and, due to their elevated readability scores, these could be too difficult for a patient to read.

## Introduction

ChatGPT (OpenAI, San Francisco, CA, USA) is a free, large language model AI chatbot. It was launched on 30 November 2022 and by April 2023 it was visited an average of over 60 million times per day. ChatGPT has natural language processing capabilities that enable it to be trained in the auto-completion of sentences and ideas. Given the word “glaucoma,” these models may predict the next word to be “open angle” or “angle closure” based on the statistical parameters learned from prior training data sets. ChatGPT has gained traction due to its unprecedented ability to generate human-like language and respond to a massive range of inputs (1).

Approximately 130 million Americans lack proficiency in literacy, with a reading level below the sixth grade (2). The average American reads material of a level between the seventh and eighth grades (2, 3). Health literacy is a recognized problem for most Americans (2–4). For patients using ChatGPT, health literacy may be a problem (1, 5, 6). ChatGPT generates text in fully formed paragraphs which may be difficult for the average patient, who has a sixth- to eighth-grade reading level.

Glaucoma is the leading cause of blindness in America (7). Its associated visual loss is painless, progressive, and can remain undetected for years. The medication regimen may involve multiple instillations of eye drops at different times of the day, leading to non-compliance (8–10).

Glaucoma is a chronic disease that requires medication adherence and an understanding of the risks and benefits of treatment. Glaucoma patients with poor education and a low socioeconomic status have worse outcomes than those with better education and a higher socioeconomic status who can better appreciate the glaucoma treatment paradigms (10–12).

Poor patient compliance has led to poor visual field outcomes and the eventual deterioration of vision (9, 10). We sought to discover if ChatGPT could be a source of patient education for our glaucoma patients, and if it is accurate and understandable (13). We used the American Academy of Ophthalmology’s website, AAO.org, an education interface for the public that was created by board-certified ophthalmologists in America, as a reference point for the questions/topics inputted into ChatGPT (14).

In our study, we delve into the connection between ChatGPT and the necessary reading levels for glaucoma literacy. Owing to the absence of patient subjects or data, Institutional Review Board approval was not required.

## Methods

In the section of the AAO’s website named “Public & Patients,” which leads to the “What is Glaucoma” page (13), we found a series of eight questions/topics to input into ChatGPT. The bot’s responses were compared with the video responses on the AAO’s website, which were then transcribed into text. We chose to use version 3.5 of ChatGPT because it is free to use.

Eight questions/topics taken from the AAO’s “Guide to Glaucoma” (13) were used: (1) “What is glaucoma?”; (2) “What causes glaucoma?”; (3) “Types of glaucoma”; (4) “What is angle-closure glaucoma?”; (5) “What are common glaucoma symptoms?”; (6) “Who is at risk for glaucoma?”; (7) “Glaucoma diagnosis”; and (8) “Glaucoma treatment”.

The AAO's website shows the following keywords as bolded and underlined:

- Question/topic 1: aqueous humor, trabecular meshwork, drainage angle, over age 60;
- Question/topic 2: aqueous humor, IOP (pressure in the eye), drainage angle, optic nerve;
- Question/topic 3: open-angle glaucoma, closed-angle/narrow-angle glaucoma, iris, acute attack, “suddenly blurry” halos around lights, severe eye pain, headache, nausea/vomiting, red eye, rainbow halos around lights, ophthalmologist;
- Question/topic 4: narrow/shallow angle, pressure increase;
- Question/topic 5: regular eye exams, ophthalmologist, angle-closure glaucoma, decreased/blurred vision, severe pain in eye/forehead, redness of the eye, seeing rainbows or halos, headache, nausea, vomiting, normal-tension glaucoma, eye pressure, ocular hypertension, pigment dispersion syndrome (PDS);
- Question/topic 6: over age 40, family history, race/African American/Asian/Hispanic, high eye pressure, farsighted, nearsighted, eye injury, long-term steroid use, thin corneas in the center, thinning of the optic nerve, diabetes, migraines, high blood pressure, poor blood circulation, other health problems affecting the whole body, ophthalmologist;
- Question/topic 7: complete eye exam, measure eye pressure, inspect eye drainage angle, examine optic nerve for damage, test peripheral/side vision, picture or computer measurement of optic nerve, measure thickness of cornea, no symptoms in its early stages, silent thief of sight, ophthalmologist;
- Question/topic 8: eye drop, glaucoma medication, stinging or itching sensation, red eyes or red skin around eyes, changes in pulse or heartbeat, changes in energy level, changes in breathing (asthma), dry mouth, blurred vision, eyelash growth, changes in eye color and skin around eyes/eyelid appearance, laser surgery, trabeculoplasty, iridotomy, operating room surgery, trabeculectomy, sclera, conjunctiva, glaucoma drainage devices, glaucoma drainage implant, cataract surgery, eye’s natural lens, narrow angles, ophthalmologist.

For the determination of reading level, we used the following readability formulas (14):

1) Gunning Fog Index


i 0.4×((words/sentences) + 100×(complexWords/words))

2) SMOG Index


ii 1.0430×sqrt (30×complexWords/sentences ) + 3.1291

3) Dale–Chall readability formula

These formulas evaluate the reading level of a text according to its word count and comprehension difficulty. Their internal algorithm defines a “difficult word” as any word outside of a predetermined list of 3,000 words (15). To use the Dale–Chall readability formula, we were directed to a different website called readabilityformulas.com, which then computed the Dale–Chall Score (DCS).

First, we calculated the raw score using the following equation:


Raw score = 0.1579×%difficult words + 0.0496×avg sentence length(words)

If the percentage of “difficult” words exceeded 5%, a Dale–Chall adjusted score was then calculated, and we used this score in our research paper


Adjusted score = raw score + 3.6365

To score the reading level, we used the Flesch–Kincaid readability test, originally used by the US Navy and Pennsylvania Insurance Department and Florida Office of Insurance Regulation for contracts (14–16), but which is now widely used (15, 16). These test scores are based on two factors, that is, the average sentence length and the average number of syllables per word: (14)


0.39×(words/sentences) + 11.8×(syllables/words)−15.59

The resulting score indicated the reading grade level needed to understand the written sentences. The transcribed English language responses were pasted into WebFx.com (14) to obtain the reading levels.

To determine the word count, we employed Google Document tools. The keywords from the answers on the AAO’s website were compared with those in the responses produced by ChatGPT (**Table 1**). In addition, important clinical concepts were selected by two ophthalmologists (GW and DAL) (**Table 1**). A total of 86 terms were compiled for the eight questions/topics taken from the AAO’s website.

**Table 1:** Selected Keywords: ChatGPT responses vs AAO website text.

Question #	AAO Terms	ChatGPT	AAO	ChatGPT Total	AAO Total
1	Aqueous humor		✓	0/4	4/4
	Trabecular Meshwork		✓		
	Drainage angle		✓		
	Over age 60		✓		
2	Aqueous humor	✓	✓	3/4	4/4
	IOP (Pressure in the eye)	✓	✓		
	Drainage angle		✓		
	Optic nerv	✓	✓		
3	Open-angle glaucoma	✓	✓	5/12	12/12
	Closed-angle/narrow-angle glaucoma		✓		
	Angle-closure glaucoma	✓	✓		
	Iris	✓	✓		
	Acute attack		✓		
	"Suddenly blurry" halos around lights	✓	✓		
	Severe eye pain	✓	✓		
	Headache		✓		
	Nausea/vomiting		✓		
	Red eye		✓		
	Rainbow halos around lights		✓		
	ophthalmologist*		✓		
4	Narrow/shallow angle		✓	1/2	2/2
	Pressure Increase	✓	✓		
5	Regular eye exams	✓	✓	4/14	14/14
	ophthalmologist**		✓		
	Angle-closure glaucoma		✓		
	Decreased/blurred vision	✓	✓		
	Severe pain in eye/forehead	✓	✓		
	Redness of the eye		✓		
	Seeing rainbows or halos		✓		
	Headache	✓	✓		
	Nausea		✓		
	Vomiting		✓		
	Normal tension glaucoma		✓		
	Eye pressure		✓		
	Ocular hypertension		✓		
	Pigment Dispersion Syndrome (PDS)		✓		
6	Over age 40		✓	10/16	16/16
	Family History	✓	✓		
	Race/African American/Asian/Hispanic	✓	✓		
	High eye Pressure	✓	✓		
	Farsighted		✓		
	Nearsighted	✓	✓		
	Eye injury	✓	✓		
	Long term steroid use	✓	✓		
	Thin corneas in the center	✓	✓		
	Thinning of optic nerve		✓		
	Diabetes	✓	✓		
	Migraines	✓	✓		
	High blood pressure	✓	✓		
	Poor blood circulation		✓		
	Other health problems affecting whole body		✓		
	ophthalmologist*		✓		
7	Complete eye exam	✓	✓	7/10	10/10
	Measure eye pressure	✓	✓		
	Inspect eye drainage angle	✓	✓		
	Examine optic nerve for damage	✓	✓		
	Test peripheral/side vision	✓	✓		
	Picture or computer measurement of optic nerve	✓	✓		
	Measure thickness of cornea	✓	✓		
	No symptoms in its early stages		✓		
	Silent thief of sight		✓		
	ophthalmologist***		✓		
8	Eye drop	✓	✓	4/24	24/24
	Glaucoma medication	✓	✓		
	Stinging or itching sensation		✓		
	Red eyes or red skin around eyes		✓		
	Changes in pulse or heartbeat		✓		
	Changes in energy level		✓		
	Changes in breathing (asthma)		✓		
	Dry mouth		✓		
	Blurred vision		✓		
	Eyelash growth		✓		
	Changes in eye color and skin around eyes/eyelid appearance		✓		
	Laser surgery	✓	✓		
	Trabeculoplasty		✓		
	Iridotomy		✓		
	Operating room surgery		✓		
	Trabeculectomy	✓	✓		
	sclera		✓		
	conjunctiva		✓		
	Glaucoma drainage devices		✓		
	glaucoma drainage imlpant		✓		
	Cataract surgery		✓		
	Eye's natural lens		✓		
	Narrow angles		✓		
	ophthalmologist****		✓		
**Total Keyword Appearance**				34/86	86/86
**Ophthalmologist mentioned in number of times**				0	26

* The term “ophthalmologist” was mentioned 1 time by the AAO website text.

** The term “ophthalmologist” was mentioned 7 times by the AAO website text.

*** The term “ophthalmologist” was mentioned 3 times by the AAO website text.

**** The term “ophthalmologist” was mentioned 14 times by the AAO website text.

## Results

The average reading grade levels of the answers on the AAO’s website and the responses produced by ChatGPT, determined using the Flesch–Kincaid test, were grade 9.4 ± 3.5 and grade 12.5 ± 1.80, respectively (*p* ≤ 0.0384) (**Table 2**). For all the reading metrics, the average reading grade level of the ChatGPT responses was higher than that of the answers on the AAO’s website. The *p*-values, all of which were less than 0.05, are listed in **Table 2**. Notably, for question 6 (risk factors for glaucoma), the answer on the AAO’s website had a higher reading grade level according to the four reading metrics. This answer contained words such as “farsighted,” “nearsighted,” “steroid medications,” “corneas,” “optic nerve,” diabetes”, and “blood circulation,” which are medical terms and not part of the vocabulary of the lay public. The term “eye doctor” appears once in the ChatGPT response to question 7. On the AAO’s website, the term “ophthalmologist” is mentioned 26 times in total in the answers to questions 3, 4, 5, 6, 7, and 8: 23 times in the text and 3 times in the videos (**Table 1**). For the results obtained using the Flesch–Kincaid test, a larger number correlates with a higher reading grade level (**Table 2**).

**Table 2:** Grade levels of the text produced in response to Questions (Qn)/topics 1–8 determined using the Flesch–Kincaid test, Gunning Fog Index, SMOG Index, and Dale–Chall readability formula.

Question #	Flesch-Kincaid Grade Level		Gunning Fog Scorer		SMOG Index		Dale-Chall-Score*	
	ChatGPT	AAO	ChatGPT	AAO	ChatGPT	AAO	ChatGPT	AAO
1	13	7.9	16.0	9.4	11.6	7.5	9.4	8.3
2	13.4	5.7	15.7	7.7	11.4	5.8	8.7	7.1
3	11.6+	7.5	14.7	9.3	10.8	6.4	9.2	7.7
4	15.6++	7.7	18.4	10.2	13.1	7.2	10.8	7.3
5	13.8	10.8	17.7	12.8	12.7	9.3	8.5	9.2
6	10.3	16.9+++	14.1	19.0	10.3	13.3	8.5	8.7
7	13	11.1	16.1	13.1	11.9	9.8	8.5	7.7
8	10.1	7.4	13.3	9.9	9.8	7.5	8.7	7.1
**Average**	12.5	9.4	15.8	11.4	11.5	8.4	9.0	7.9
**Standard deviation**	1.8	3.5	1.7	3.6	1.1	2.4	0.8	0.8
***p*-value**	0.0384		0.00788		0.00531		0.0108	

Flesch–Kinkaid test, Gunning Fog Index, SMOG Index: Years of education = grade level.

*Dale–Chall scores: score 9.0–8.0 = grades 11–12; score 9.0–10.0 = college; score “ 10.1 = college graduate.

+ 11.6 = junior year in high school.

++ 15.6 = junior year in college.

+++ 16.9 = senior year college/graduate school level.

*Dale–Chall scores: score 9.0–8.0 = grades 11–12; score 9.0–10.0 = college student; score ≥10.1 = college graduate.

Each ChatGPT (CG) response and the text on the AAO’s website were scored for the correlation of keywords. For the ChatGPT responses, the final score was 34 out of 86, compared with 86 out of 86 for the text on the AAO’s website. In terms of word counts, the responses on the AAO’s website and those produced by ChatGPT had similar word counts when averaged over the eight questions/topics. For five out of eight questions/topics, the responses produced by ChatGPT had a greater word count than the answers on the AAO’s website (**Table 3**)

**Table 3:** Word count for ChatGPT responses and the AAO website text.

Question #	Word Count	
	ChatGPT	AAO
1	119	81
2	182	125
3	235	274
4	167	33
5	163	352
6	217	97
7	232	121
8	209	947
**Average**	190.5	253.8
**Standard Dev**	40.1	299.5
**P-value**	0.571	

The same eight questions/topics were inputted into ChatGPT on 4 January 2023 and again on 5 May 2023, and the responses remained the same.

## Discussion

During the COVID-19 pandemic, many patients resorted to self-isolation or were hesitant to visit their eye doctors. Consequently, their family members turned to the internet for patient information; however, the information found on the internet can be incorrect (17–21). The introduction of ChatGPT on 30 November 2022 fulfilled an unmet need, drawing users from all parts of the world (17–19).

Our study was focused on exploring ChatGPT’s ability to engage with glaucoma patients. It is estimated that 4 million Americans have this disease, with an additional 2.4 million undiagnosed cases (17).

ChatGPT has been trained using internet data from approximately 2021, and its popularity lies in its ease of use. In the space of one week in 2021, it gained millions of users. The literature has discussed the need for patient education for individuals with chronic conditions such as diabetes, for which certified diabetes educators play a crucial role (9). Although there are no certified ophthalmic educators, certified ophthalmic technicians could help with the education of our glaucoma patients. These ophthalmic technicians are paid employees, whereas ChatGPT is free. The use of ChatGPT would achieve cost savings for our office budgets, but the responses provided by it are not accurate enough to function as standalone solutions that address the gaps in glaucoma patients’ education (22, 23).

Although ChatGPT has great potential to contribute to patient education, its responses seldom contain the terms “eye doctor” or “ophthalmologist”. There is a distinction between the training and surgical knowledge and expertise of an eye doctor (i.e., an optician or optometrist) and that of a board-certified physician ophthalmologist. When prompted with the term “glaucoma,” ChatGPT’s predictions were based on statistical parameters learned from prior training data sets and internet data from 2021, meaning that its responses are potentially missing newer information related to innovative medications and techniques for glaucoma treatment. In addition, the reliability of the training data sets used for chatbots such as ChatGPT remains unclear, particularly if data sets rely heavily on sources such as Wikipedia and social media platforms that may not explicitly mention “ophthalmology” or “ophthalmologist” in the context of glaucoma (5, 18, 19, 22).

Search engine optimization of the AAO’s website may depend on the user’s location and search history, potentially affecting the visibility of the website when searching for glaucoma. Furthermore, the AAO’s website may not have utilized specific coding practices to associate “ophthalmology” with “glaucoma,” thus limiting chatbots and other search engines from recognizing the connection between these terms.

Although ChatGPT is trained in medical literature, articles written by ophthalmologists for ophthalmologists do not commonly include phrases such as “see an ophthalmologist” in the context of glaucoma. As a consequence, chatbots will not generate such phrases with the word “ophthalmologist.”

Although reading-level assessment tools are not perfect, we used those commonly used by the military and state insurance bureaus. ChatGPT’s text responses require reading comprehension at an 11th-to-12th-grade reading level, whereas the average American reads at a 7th-to-8th-grade level (2, 3). This discrepancy poses a challenge for patients with lower health literacy and may affect their understanding of treatment relevance and vision loss prevention. Improved adherence to glaucoma eye drops is closely associated with higher health literacy, socioeconomic status, and education level (10–12). In order to elicit a simpler explanation of glaucoma, one could preface their instruction to ChatGPT with the phrase "Tell me like I am in fifth grade." However, for the purpose of the study, we consciously used the exact questions found on the AAO's website when preparing our data.

In comparing the responses produced by ChatGPT with the answers on the AAO’s website, we found that the latter were at a lower reading grade than high school level, although they still exceeded the comprehension level of seventh- or eighth-graders. The inclusion of videos on the AAO’s website enhances its accessibility and clarity. Conversely, ChatGPT’s responses may contain errors of omission, potentially leading patients to disregard their glaucoma symptoms or underestimate the urgency of seeking specialized care (**Table 1**). The chatbot’s responses do not emphasize that angle closure can rapidly cause irreversible vision loss or the urgency of consulting an eye care specialist, ophthalmologist, or emergency room physician (**Table 1**). This is consistent with ChatGPT’s programming, which is to provide descriptions but not “make decisions” (17). Repeated questioning of ChatGPT led to similar answers, with minimal changes in the content. However, if the subsequent queries were different each time, the answers would be more variable. For the purposes of this study, we used the same wording as in the AAO glaucoma questions for the queries inputted into ChatGPT.

The availability of large language models such as ChatGPT has ushered in a new era in which physicians can leverage AI for clinical decision-making and limited patient education (13, 17, 19). Other large language models, such as BARD and PaLM2, also exist (20, 21). Both of these were created by Google’s team of AI developers. BARD was released to a select audience in March 2023 and April 2023 but opened to the general public in mid-August 2023 in the USA, with a multilingual capability of 40 languages (24–27). The original version of BARD launched in February 2023 contained bugs, which led to the closure of the app 24 hours later. PaLM2 is another Google product with 140 languages in its algorithms and a greater ability for “deductive reasoning.” There are plans for PaLM2 to intersect with technical fields, such as medicine, health, and programming languages (21). The PaLM2 family of algorithms also feeds into BARD (20, 21). For now, the free version 3.5 of ChatGPT continues to be updated. The field of AI is quickly changing, and its major players will continue to make their chatbots more responsive to the public’s needs.

In the near future, ophthalmology residency and fellowship programs may utilize chatbots to enhance trainees’ clinical reasoning skills through generative case reports, similar to oral board examination questions. As physicians, we must be aware of the capabilities and limitations of AI-mediated chatbots, as they may generate inaccurate or biased results (17–21). It is crucial for physicians to recognize the influence of their written “phrases” and “word associations,” as these are used in the training of chatbots by their software programmers. In addition, the frequent inclusion of the phrase “see an ophthalmologist” in the software codes of ophthalmic websites and web journals may be needed to ensure the inclusion of the word “ophthalmologists” in the training algorithms of AI-mediated chatbots.

## Data availability statement

The original contributions presented in the study are included in the article/supplementary material. Further inquiries can be directed to the corresponding author.

## Author contributions

GW: Conceptualization, Data curation, Investigation, Methodology, Writing – original draft, Writing – review & editing, Formal Analysis, Validation, Visualization. DL: Conceptualization, Data curation, Investigation, Methodology, Writing – original draft, Writing – review & editing, Validation, Visualization, Formal Analysis. WZ: Conceptualization, Data curation, Investigation, Methodology, Writing – original draft, Writing – review & editing, Validation, Formal Analysis, Visualization. AW: Conceptualization, Data curation, Investigation, Methodology, Writing – original draft, Writing – review & editing, Validation, Visualization, Formal Analysis. SS: Conceptualization, Data curation, Investigation, Methodology, Writing – review & editing, Validation, Visualization, Formal Analysis.
